# Herbivory by a Phloem-Feeding Insect Inhibits Floral Volatile Production

**DOI:** 10.1371/journal.pone.0031971

**Published:** 2012-02-23

**Authors:** Martin Pareja, Erika Qvarfordt, Ben Webster, Patrick Mayon, John Pickett, Michael Birkett, Robert Glinwood

**Affiliations:** 1 Departmento de Entomologia, Universidade Federal de Lavras, Lavras, Brazil; 2 Department of Ecology, Swedish University of Agricultural Sciences, Uppsala, Sweden; 3 Rothamsted Research, Department of Biological Chemistry, Harpenden, United Kingdom; French National Centre for Scientific Research, Université Paris-Sud, France

## Abstract

There is extensive knowledge on the effects of insect herbivory on volatile emission from vegetative tissue, but little is known about its impact on floral volatiles. We show that herbivory by phloem-feeding aphids inhibits floral volatile emission in white mustard *Sinapis alba* measured by gas chromatographic analysis of headspace volatiles. The effect of the Brassica specialist aphid *Lipaphis erysimi* was stronger than the generalist aphid *Myzus persicae* and feeding by chewing larvae of the moth *Plutella xylostella* caused no reduction in floral volatile emission. Field observations showed no effect of *L. erysimi*-mediated floral volatile emission on the total number of flower visits by pollinators. Olfactory bioassays suggested that although two aphid natural enemies could detect aphid inhibition of floral volatiles, their olfactory orientation to infested plants was not disrupted. This is the first demonstration that phloem-feeding herbivory can affect floral volatile emission, and that the outcome of interaction between herbivory and floral chemistry may differ depending on the herbivore's feeding mode and degree of specialisation. The findings provide new insights into interactions between insect herbivores and plant chemistry.

## Introduction

Volatile organic compounds emitted from plants mediate an array of ecological interactions. They play important roles in plant defence as herbivore deterrents [1], [2], [3] and in the attraction of predators and parasitoids to herbivore damaged plants [for reviews see 4], [5]. Furthermore, volatiles emitted from flowers play a vital role in mediating the mutualism between plants and their pollinators [6]. For many years pollination ecology and plant defence were studied separately [7], as adaptations to distinct and independent selective pressures. However, there have been advances in the integration of pollination and plant defence [8], in particular highlighting how flower traits are often evolutionarily constrained by selective pressures acting on plant defence [9]. It has even been argued that, in evolutionary terms, floral volatiles were originally involved in other aspects of plant behaviour such as defence [10]. Floral volatiles have the potential to mediate not only interactions of plants with pollinators, but a series of direct and indirect interactions with other organisms such as herbivores, predators and microorganisms [11].

There is growing awareness of the ecological importance of the connection between the chemistry of pollination and that of plant defence [12], [13], [14] but, despite the extensive evidence of the effects of different types of herbivore on the production of volatiles by vegetative tissues, only a handful of studies have explored the relationship between the emission of floral volatiles and damage to vegetative tissues. Effmert et al. [15] found that, although caterpillar-damaged vegetative tissues emitted herbivore-induced volatiles, there was no effect of chewing damage on floral volatiles. Theis et al. [16] found increased volatile terpenoid emission from male flowers after mechanical simulation of chewing. Kessler et al. [17] found that damage by *Manduca* spp. caterpillars caused reduced emission of the floral volatile benzyl acetone along with major changes in flower phenology.

The damage in the cited studies was caused by leaf chewing, which is known to elicit strong jasmonate (JA) signalling and reduced levels of salicylate (SA)-mediated responses [18], [19], [20]. Phloem feeding herbivores, such as aphids and whiteflies, often elicit SA-mediated signalling [21], [22], [23]. Biosynthesis of SA is related to the shikimate pathway that leads to phenylalanine and phenolic compounds via cinnamic acid [24]. Benzenoid compounds, derived via cinnamic acid metabolism [25], are prominent in floral scents [26], so damage by phloem feeders is predicted to have different effects on floral volatile emissions to those caused by chewing insects. Even within the phloem-feeding guild, plant responses may vary depending on the degree of specialisation of the herbivore [27], and evidence is emerging that phloem-feeding aphids may be able to actively manipulate plant defences [28].

In a study of aphid-induced plant volatile emission, we tested the effect of feeding on vegetative tissue by the mustard aphid *Lipaphis erysimi* on the emission of floral volatiles in white mustard, *Sinapis alba* (Brassicaceae). Surprisingly, instead of inducing production of floral volatiles, the aphid caused a substantial reduction in emissions. We designed the current study to explore this effect and to address the following hypotheses: (i) insect herbivore feeding on vegetative tissue affects the identity and/or quantity of volatile emissions from flowers, (ii) the impact of herbivory depends on the herbivore's feeding mode (aphids compared with larvae of the diamondback moth *Plutella xylostella*) and on the degree of specialisation (*L. erysimi* compared with the highly polyphagous peach-potato aphid *Myzus persicae*) (iii) herbivore-induced changes in floral volatile emission disrupt pollinator attraction and reduce chemical apparency to the herbivores' natural enemies. The results demonstrate for the first time that a phloem-feeding herbivore can affect the volatile chemistry of flowers.

## Results

### A specialist aphid inhibits floral volatiles more strongly than a generalist aphid but a chewing herbivore has no effect

**Figure 1:**
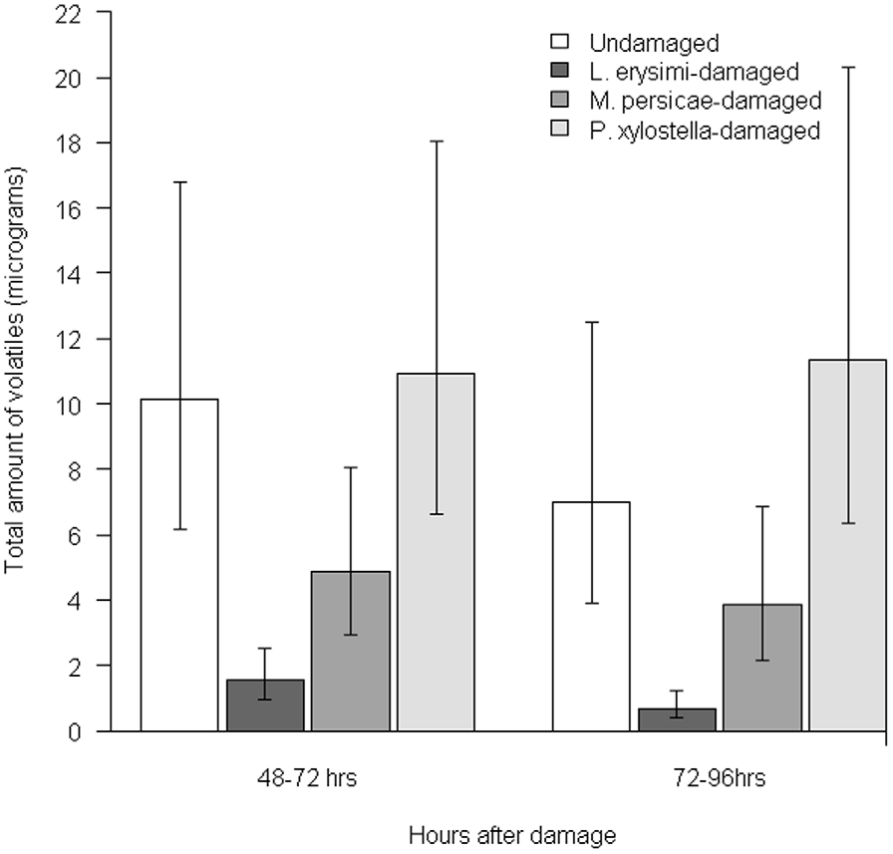
Floral volatiles from *Sinapis alba* infested with different herbivores. Figures represent total volatiles (micrograms, mean ± 95% confidence interval). Volatiles were collected in two periods: 24–72 h after the start of herbivory by aphids *Lipaphis erysimi*, *Myzus persicae* or larvae of the moth *Plutella xylostella* and 72–96 h after the start of herbivory. Aphids caused a significant reduction in floral volatile emission at 24–72 h (ANOVA, F_3,12_ = 5.50, P = 0.013) and 72–96 h after infestation (ANOVA, F_3,12_ = 5.07, P = 0.017).

Emission of floral volatiles from *S. alba* was investigated in response to feeding by a specialist aphid *L. erysimi*, a generalist aphid *M. persicae* and a chewing moth larva *P. xylostella*. Volatiles emitted by flowers were collected by air entrainment and analysed by gas chromatography. To analyse changes in the floral blends after herbivore attack, a discriminant analysis using compositional data was carried out. Aphid herbivory on *S. alba* decreased floral volatile production in the periods 48–72 h after aphid infestation (F_3,12_ = 5.50, P = 0.013) and 72–96 h after infestation (F_3,12_ = 5.07, P = 0.017) (Figure 1). The decrease was more pronounced with *L. erysimi* than with *M. persicae*. Feeding by *P. xylostella* caused a slight increase in volatile production (Figure 1). The mean amounts of compounds detected in the different herbivore treatments are shown in Table 1. The discriminant analysis at 48–72 hours significantly separated floral blends of *L. erysimi*-damaged plants from those of undamaged plants (*P* = 0.012), floral blends of *L. erysimi*-damaged plants from those of *M. persicae*-damaged plants (*P* = 0.048) and floral blends of *L. erysimi*-damaged plants from those of *P. xylostella*-damaged plants (*P* = 0.016). The first root (dimension) of the discriminant analysis separated *L. erysimi*-damaged plants from the other treatments. Benzaldehyde and benzyl alcohol were the compounds most associated with *L. erysimi* damage, indicating that they were reduced, but to a lesser extent than the other benzenoids. The compounds that were most affected were *p*-anisaldehyde and methyl salicylate. The second root separated *P. xylostella*-damaged plants from *M. persicae*-damaged plants. The compounds most associated with *P. xylostella* damage were benzyl alcohol, benzyl acetate and vanillin, whereas the compound most associated with *M. persicae* damage was p-anisaldehyde.

**Table 1:** Volatile compounds in the headspace of flowers of *Sinapis alba* subjected to herbivory to vegetative parts.

Compound	Amount (ng) detected in each 24 hour period^1^							
48–72 hours after start of herbivory				72–96 hours after start of herbivory				
	UD	*L. erysimi*	*M. persicae*	*P. xylostella*	UD	*L. erysimi*	*M. persicae*	*P. xylostella*
Benzaldehyde	5400±1508	629±216	2225±1009	5462±1887	4135±1747	198±80	2273±1252	6327±2583
6-Methyl-5-hepten-2-one	-	Trace	Trace	15±9	Trace	-	-	Trace
(+)-Sabinene	-	-	Trace	13±8	-	-	-	Trace
Myrcene	9±5	20±13	37±23	72±34	8±5	19±12	14±9	35±18
(*Z*)-3-Hexenyl acetate	46±14	23±11	Trace	20±10	51±14	14±6	13±8	25±6
Benzyl alcohol	255±85	95±53	205±143	411±158	217±114	38±19	80±15	327±165
(*E*)-Ocimene	195±28	33±4	70±18	109±40	113±16	Trace	51±16	108±34
Benzyl acetate	28±14	-	-	34±10	Trace	-	-	24±17
Methyl salicylate	68±20	33±14	49±17	78±19	73±16	37±11	57±8	94±11
*p*-Anisaldehyde	4360±803	528±190	2370±586	4900±865	2566±270	118±62	1970±676	4557±812
Indole	702±232	85±17	240±102	262±162	354±195	Trace	79±32	202±62
2-Undecanone	83±20	66±8	71±16	132±37	85±16	68±14	75±19	147±32
Benzyl isothiocyanate	Trace	Trace	7±4	Trace	-	-	-	-
Vanillin	55±10	34±9	66±27	185±49	65±6	Trace	80±29	230±53
Methyl vanillin	116±91	-	102±14	14±6	96±90	-	77±60	12±8
(*E,E*)-α-Farnesene	53±20	12±8	22±8	21±13	23±12	-	19±14	15±9

Undamaged (UD), subjected to damage by the aphids *Lipaphis erysimi*, *Myzus persicae* or *Plutella xylostella* caterpillars, 48–72 hours and 72–96 hours after the start of damage. Means (± standard error). Compounds are in order of elution on an HP-1 GC column.

1 Mean (± s.e.) from five replicates (see Methods). Trace indicates compound was detected in a single sample. (-) indicates that the compound was not detected in any sample.

At 72–96 hours the discriminant analysis significantly separated floral blends of *L. erysimi*-damaged plants from those of undamaged plants (*P* = 0.004), floral blends of *L. erysimi*-damaged plants from those of *M. persicae*-damaged plants (*P* = 0.005) and floral blends of *L. erysimi*-damaged plants from those of *P. xylostella*-damaged plants (*P* = 0.005). The first root (dimension) of the discriminant analysis separated the *L. erysimi*-damaged plants from the other treatments. The first root again separated *L. erysimi*-damaged plants from the other treatments. During this period the compounds most affected were benzaldehyde and methyl salicylate, and the compound least affected was benzyl alcohol, indicating a compositional change in the benzaldehyde/benzyl alcohol balance between the days. The second root again separated *P. xylostella* damage from *M. persicae* damage. The compounds most related to *P. xylostella* damage were benzyl alcohol, benzyl acetate and vanillin, and those most related to *M. persicae* damage were benzaldehyde and methyl salicylate.

### Inhibition of floral volatiles is dependent on the number of feeding aphids

**Figure 2:**
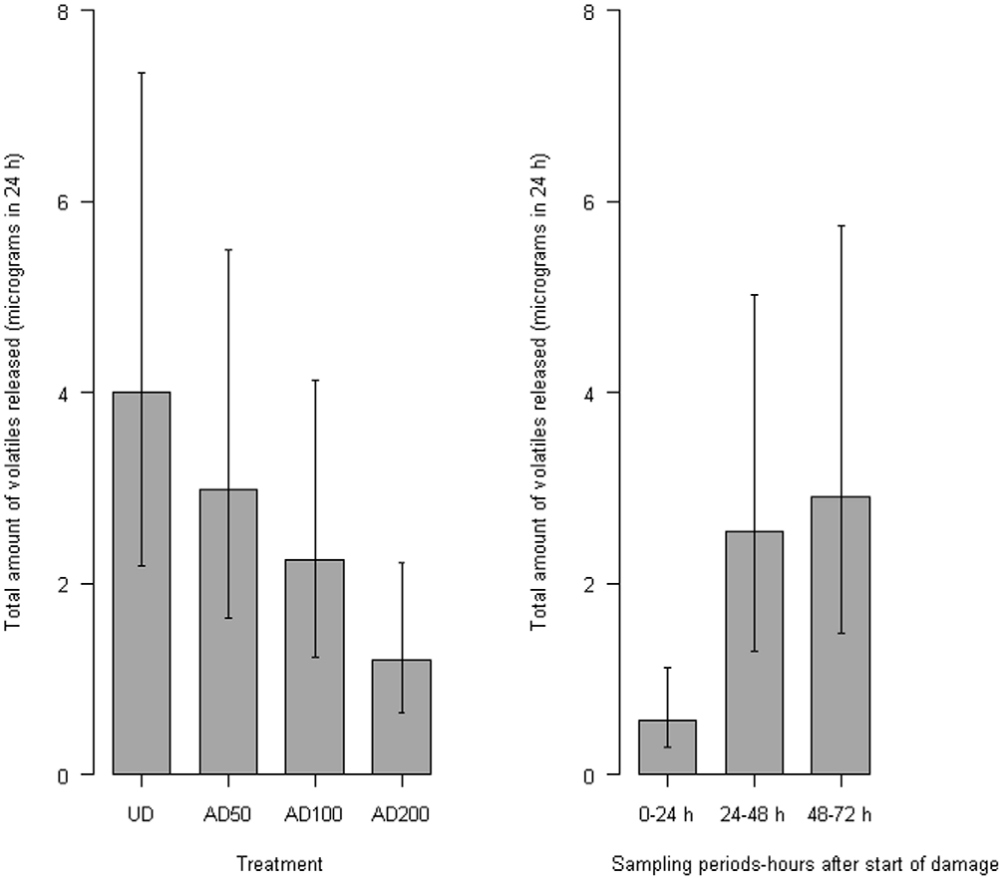
Floral volatiles from *Sinapis alba* infested with different numbers of aphids. Total amount of volatiles (micrograms, mean ± 95% confidence interval) emitted from *Sinapis alba* flower heads of undamaged plants and plants subjected to damage by either 50 (AD50), 100 (AD100) or 200 (AD200) *Lipaphis erysimi* over a 72 h period. Left: total for each aphid density treatment averaged over all time periods. Right: total for each time period averaged over different aphid density treatments. Aphid density had a negative effect on volatile production by *S. alba* (ANOVA, F_1,17_ = 7.88, P = 0.012) and there was no interaction between aphid density and time (ANOVA, F_2,44_<0.01, P = 0.997).

To test how the density of *L. erysimi* and time after infestation affect the reduction in floral volatile production observed in the previous experiment, the density of *L. erysimi* on plants at the beginning of flowering was manipulated to give densities of 50, 100 and 200 aphids per plant as well as an uninfested control. There was a large overall increase in volatile release from day one to day three (F_2,44_ = 11.31, P<0.001) (Figure 2). *L. erysimi* density had a negative effect on volatile production by *S. alba* (F_1,17_ = 7.88, P = 0.012) (Figure 2). There was no interaction between *L. erysimi* density and time (F_2,44_<0.01, P = 0.997), indicating that the decrease due to aphid infestation begins during the first day, and remains constant during the next three days.

### Aphid feeding after the start of flowering does not inhibit floral volatile production

The initial experiments tested the effect of aphid infestation before the start of flowering, however in this experiment the first flowers were allowed to open before aphids were placed on the plant. *L. erysimi* infestation after floral volatile release had begun did not reduce volatile emission (F_1,3_ = 0.466, P = 0.544). There was a statistically significant increase in volatile release over time (F_1,8_ = 7.11, P = 0.029), but this increase was similar in uninfested and *L. erysimi*-infested plants (no statistically significant interaction, F_1,8_ = 2.81, P = 0.132). Finally, the amount of volatiles released during the first day (prior to aphid infestation) was significantly and positively related to the amount released on subsequent days (F_1,3_ = 12.47, P = 0.039).

### Inhibition of floral volatiles is not due to reduced number of flowers

Feeding by *L. erysimi* had no significant effect on the number of flowers produced by *S. alba* (Le-damaged: mean number of flowers 11.1, se = 0.8; UD: mean 11.4 se = 0.8; Treatment effect F_7,11_ = 0.18, P = 0.679; block effect F_7,11_ = 0.92, P = 0.526). Thus the reduction in floral volatiles did not result from a reduction in the number of flowers emitting scent.

### Aphids induce volatile emission in vegetative tissues of *S. alba*

To investigate and the effect of aphid feeding on volatile emission from the vegetative parts of *S. alba*, volatiles were collected from stalks and leaves infested with *M. persicae* and *L. erysimi* at the same growth stage as for the floral volatile collections. The volatile blends were analysed by discriminant analysis.

The mean amounts of compounds detected in the different herbivore treatments are shown in Table 2. The total amount of volatiles did not differ among treatments (*F_2,14_* = 1.86, *P* = 0.193) but the compositional discriminant analysis revealed differences in the relative contributions of compounds to each blend. There were significant differences between the blends of *M. persicae* damaged plants and those of undamaged plants (*P* = 0.040). The first root of the discriminant analysis separated *M. persicae*-damaged plants from undamaged plants, with *L. erysimi*-damaged plants being intermediate between the two. The compounds most associated with *M. persicae*-damaged plants were (*Z*)-3-hexen-1-ol, 6-methyl-5-hepten-2-one, (*RS*)-limonene and benzyl isothiocyanate. Those most associated with undamaged plants were heptyl acetate and nonyl acetate. The second root of the discriminant analysis separated *L. erysimi*-damaged plants from the other two treatments. The compounds most associated with *L. erysimi* damage were 6-methyl-5-hepten-2-one, 2-ethylhexyl acetate and 2-tridecanone. Those associated with the other two treatments were 2-undecanone and benzyl isothiocyanate.

**Table 2:** Volatile compounds in the headspace of vegetative parts of *Sinapis alba*.

Compound	Amount (ng) detected in 24 hour period^1^Aphid treatment		
	Undamaged	*L. erysimi*	*M. persicae*
3-Butenyl isothiocyanate	-	-	2.46±1.30
6-Methyl-5-hepten-2-one	-	2.75±1.53	10.10±4.59
(*Z*)-3-Hexenyl acetate	2.70±1.37	21.95±16.59	32.02±15.22
Hexyl acetate	6.05±1.69	5.58±1.66	7.57±2.28
(*RS*)-Limonene	-	-	1.97±1.04
Heptyl acetate	2.80±1.61	1.96±0.65	1.76±0.61
2-Ethylhexyl acetate	13.16±2.85	8.44±1.89	7.67±2.46
Methyl salicylate	-	0.44±0.32	1.52±0.63
Nonyl acetate	2.11±0.63	1.66±0.54	0.82±0.48
2-Undecanone	3.42±1.26	2.96±1.14	1.87±0.89
Benzyl isothiocyanate	-	-	12.83±8.43
(*E,E*)-α-Farnesene	-	-	1.12±0.99
TMTT^2^	-	-	1.47±0.55
2-Tridecanone	-	0.30±0.23	0.07±0.04
Total	30.25±2.01	46.76±16.65	84.55±33.48

Plants subjected to herbivory by the aphids *Lipaphis erysimi* or *Myzus persicae*. Volatiles collected 48–72 hours after the start of damage. Means (± standard error). Compounds are in order of elution on an HP-1 GC column.

1 Mean (± s.e.) from six replicates (see Methods). (-) indicates that the compound was not detected.

2 4,8,12-Trimethyl-1,3,7,11-tridecatetraene.

### Pollinator visits to *S. alba* are unaffected by aphid feeding

To investigate whether aphid inhibition of floral volatile emission affects attraction of pollinators to *S. alba* flowers, plants were infested with aphids in the laboratory and then placed in the field, and the number of pollinator visits recorded. Flowers were visited by small numbers of several common pollinators including solitary bees, syrphids and bumblebees. The total number of visitors did not differ between treatments (F_2,35_ = 0.02, P = 0.985): UD 14.5±3.1, Le 13.9±3.1 and Mp: 14.5±3.1 (mean number of visits per day ± s.e.).

### Aphid inhibition of floral volatiles does not impact olfactory orientation by aphid natural enemies

Aphid natural enemies use aphid-induced volatiles in host location and can use flower resources as food. Olfactometry was used to test the relative importance of volatile cues from flowering and vegetative plant parts for the aphidophagous ladybird *Coccinella septempunctata* and the parasitoid *Diaeretiella rapae*.

**Figure 3:**
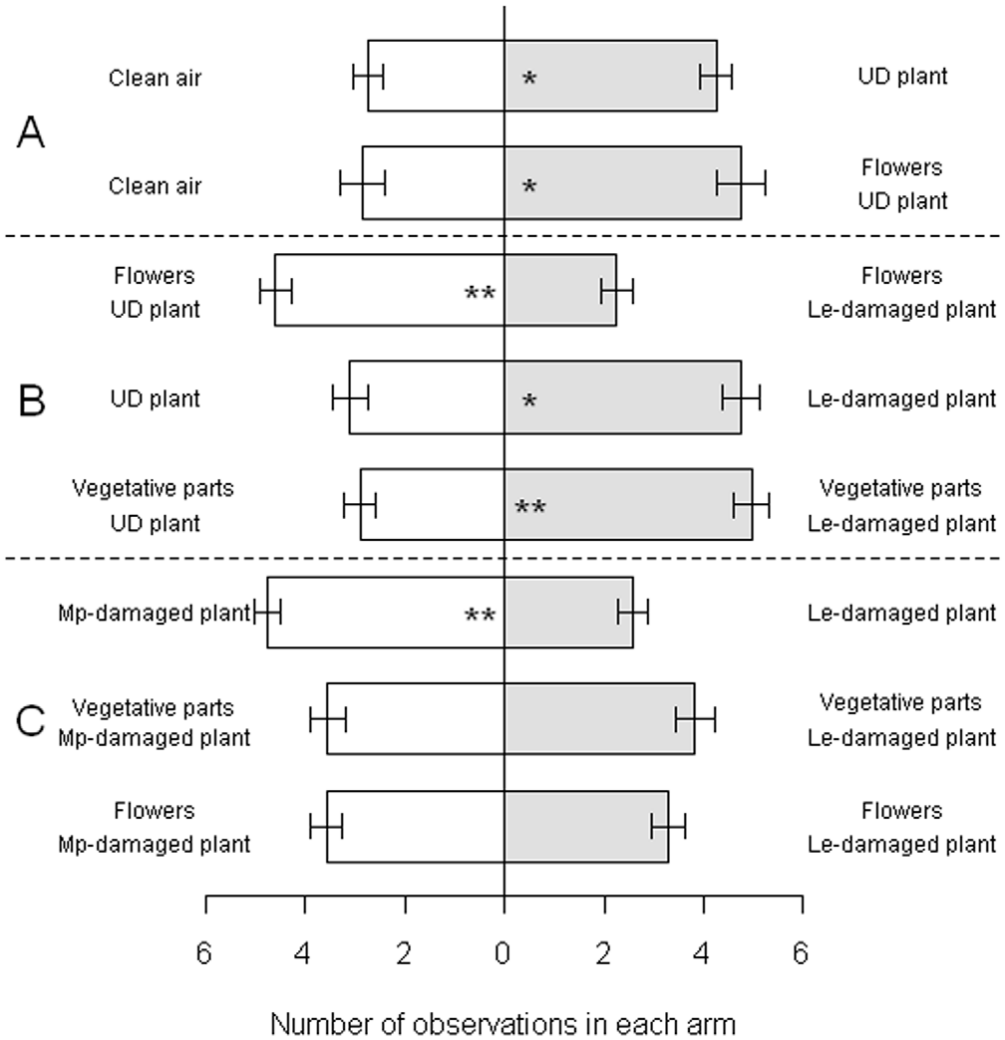
Response of ladybird *C. septempunctata* to different odour combinations in a two-way olfactometer. Figures represent mean number of observations in each olfactometer arm (± s.e.). UD: undamaged *S. alba*. Le: *L. erysimi* Mp: *M. persicae*. The different sections represent different comparisons carried out: A.) comparisons to test attraction to undamaged *S. alba*; B.) comparisons to test for attraction to *L. erysimi*-damaged plants; C.) comparisons between *L. erysimi* damage and *M. persicae* damage. * 0.05<p<0.01; ** 0.01<p<0.001.

*C. septempunctata* was attracted by the odour of undamaged (UD) flowering *S. alba* plants and by odour of *S. alba* flowers (Figure 3A). Ladybirds significantly preferred the odour of flowers from undamaged plants over that of flowers from *L. erysimi*-damaged plants. However, they preferred odour of *L. erysimi*-damaged whole plants over undamaged whole plants, and preferred vegetative parts of *L. erysimi*-damaged plants over vegetative parts of undamaged plants (Fig. 3B). Ladybirds significantly preferred odour of *M. persicae* damaged plants over *L. erysimi* damaged plants, but showed no preference between plants damaged by the different aphids when odours from only flowers or vegetative parts were compared (Fig. 3C).

**Figure 4:**
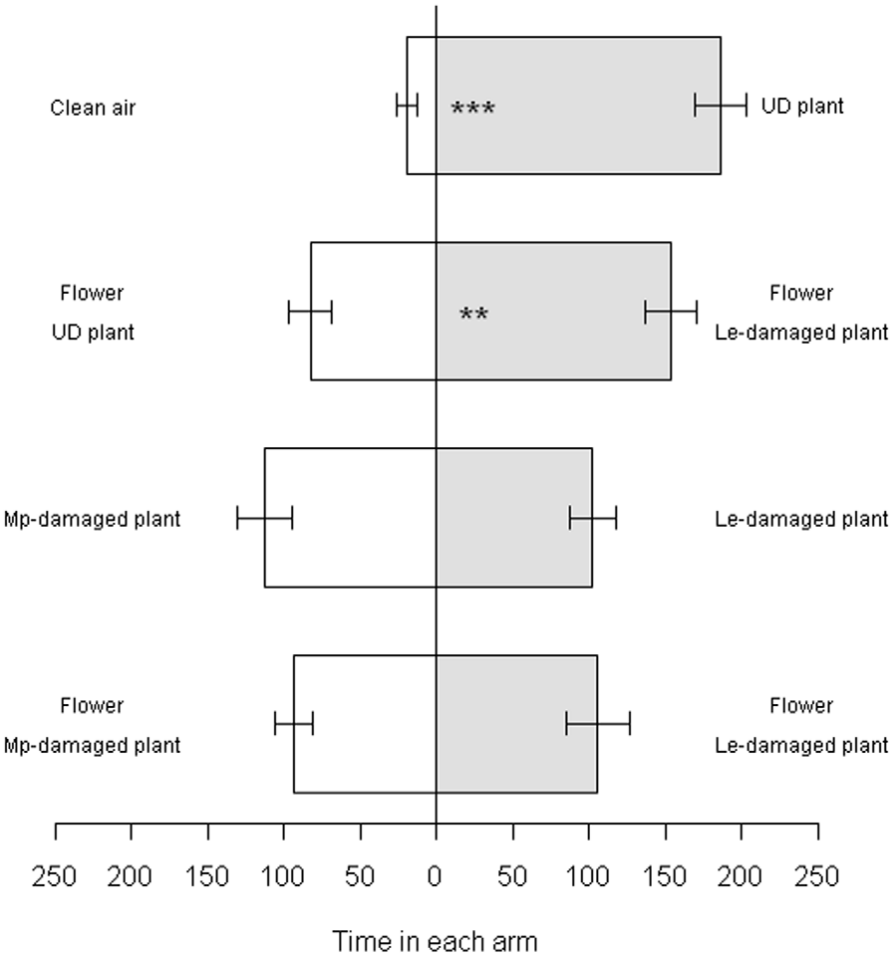
Response of aphid parasitoid *D. rapae* to odours from *S. alba* in a two-way olfactometer. Figures represent mean time spent in each arm in seconds (± s.e.). UD: undamaged flowering *S. alba*. Le: *L. erysimi*. Mp: *M. persicae*. ** 0.01<p<0.001; *** p<0.001.

*D. rapae* spent significantly more time in the odour field of undamaged flowering *S. alba* when compared with clean air, and also showed a significant preference for odour of flowers from *L. erysimi*-damaged plants over flowers from undamaged plants (Figure 4). The parasitoid showed no preference for odours from plants damaged by *L. erysimi* or *M. persicae* when odours from whole plants or flowers alone were compared (Fig. 4).

## Discussion

We found that (i) herbivory by aphids inhibits the production of floral volatiles in *S. alba*, (ii) the inhibition was not caused by a chewing herbivore and was more pronounced in a specialist aphid than in a generalist, (iii) inhibition of floral volatile production did not have a major disruptive effect on pollinator visitation or on olfactory orientation of the aphids' natural enemies. Previous studies have reported that vegetative feeding by chewing herbivores can result in floral volatile emissions that are either enhanced [16], reduced [17] or unaffected [15]. Our study is the first to report that phloem-feeding herbivory affects floral volatile emission, and shows that the outcome of interaction between herbivory and floral chemistry may differ depending on the herbivore's feeding mode and degree of specialisation.

Rather than being a consequence of herbivory-related stress in general, inhibition of floral volatiles was dependent on the identity of the herbivore. The more pronounced effect of *L. erysimi* compared with the highly polyphagous *M. persicae* may indicate that the degree of aphid specialisation is also important. Differences in the way aphids interact with host plants at the molecular and biochemical level have been found between *Brassica* specialists and generalists [27], [29], [30], and our results provide further evidence that plant responses to herbivory vary with the identity and feeding mode of the herbivore [20], [31].

We did not investigate the mechanisms underlying the suppression of floral volatile production by aphid feeding but potential mechanisms can be proposed. Stress caused by aphid damage could compromise general plant health to an extent that floral volatile production cannot be supported. This is unlikely since the relatively severe damage caused by the chewing herbivore *P. xylostella* had no negative effect. Another possibility is that aphids remove phloem sap, which could contain signalling molecules. The effect of *L. erysimi* was more pronounced than that of *M. persicae* and there is no reason to expect the specialist and generalist aphid to selectively remove phloem components, although through differences in feeding behaviour, the specialist may represent a stronger sink than the generalist. Nevertheless, the most likely explanation involves the interaction between aphids and plant defence pathways. Plant responses to phloem feeders differ from those by chewing herbivores [20]; aphids cause less damage to plant tissues and activate salicylate-mediated responses more than those mediated by jasmonate signalling [18], [22], [32]. The major compounds in the floral scent were volatile benzenoid compounds derived from the breakdown of phenylalanine by phenylalanine ammonia-lyase (PAL). This enzyme has been implicated in plant defence against aphids [33], and it is possible that aphid feeding resulted in diversion of either substrate or enzymes into induced defence and away from floral volatile production.

Piercing-sucking insects in particular have been shown to be able to evade, suppress or manipulate plant defences to their advantage [28], [32], [34], thus a specialist aphid such as *L. erysimi* may be more highly adapted for disarming specific defence pathways found in its host plant, giving rise to the differences in volatile inhibition found for the two aphid species. We hypothesize that feeding by the specialist aphid *L. erysimi* suppresses floral volatile production through modification of these interrelated defence pathways by the introduction, into the phloem, of salivary factor(s), and a role for phloem mobile chemical signalling.

A primary role for floral volatiles in *S. alba* should be attraction of insect pollinators [35]. Thus we hypothesised that the strong inhibition of floral volatiles caused by *L. erysimi* in the laboratory would negatively impact the plant's ability to attract these insects. However, in our field experiment we did not observe a reduction in the number of visits to infested plants. Visitation was generally low, possibly due to the absence of *S. alba* in the habitat. It is possible that in habitats where the plant is abundant, pollinator visitation will be higher and aphid damaged individuals will suffer a reduction in pollinator visitation. Although we did not find effects on insect pollinators here, this ecological interaction deserves to be explored further. We found that aphid feeding reduced the amounts of both major and minor components of the floral volatile blend. The major benzenoid components are often reported as pollinator attractants [e.g. 36] but even floral compounds occurring in small amounts can be important in this regard [37]. Interactions between herbivores and pollinators mediated by plant chemistry could be common in natural ecosystems and the effects could be highly dependent on the ecological context, varying with the pollinator community and availability of alternative resources.

The natural enemies of herbivores respond to changes in plant volatile emissions induced by herbivore feeding as a means of locating their prey [5]. We tested olfactory orientation of the aphidophagous ladybird *C. septempunctata* and the parasitoid *D. rapae* which use aphid-induced volatiles in host location [23], [38] and may also use flower resources as food, with the hypothesis that inhibition of floral volatiles would allow aphids to become less chemically apparent to their predators.

Ladybirds were attracted to odour of *S. alba* flowers but preferred the odour of flowers from undamaged plants over odour of flowers from *L. erysimi*-infested plants. This suggests that the ladybird is attracted to floral volatiles and that attraction may be disrupted by the effects of aphid feeding, potentially allowing *L. erysimi* to avoid detection. However, when presented with the entire plant, ladybirds were more attracted to infested plants and may have responded to the volatiles induced in vegetative tissues by aphid feeding or to the modified blend. Behavioural and/or electrophysiological responses of *C. septempunctata* have been reported to (*Z*)-3-hexen-1-ol and (*Z*)-3-hexenyl acetate [39], methyl salicylate [40], which were either induced or increased in *L. erysimi*-infested plants. Thus it appears that any reduction in ladybird attraction due to reduced floral volatile emission was overridden by volatiles associated with aphid feeding on vegetative tissues.

Ladybirds preferred odour of *M. persicae*-infested plants over *L. erysimi*-infested, possibly reflecting a preference for the generalist aphid over the *Brassica* specialist which can sequester toxic glucosinolates from the host plant [41]. There were qualitative and quantitative differences in the volatiles induced by *L. erysimi* and *M. persicae* that could potentially allow the ladybird to differentiate between the two blends. Several compounds were detected only from *M. persicae*-infested plants, including the 3-butenyl- and benzyl isothiocyanates, whereas 2-tridecanone was detected exclusively from *L. erysimi* infestation. When presented with vegetative parts or flowers alone however, ladybirds showed no preference for either aphid treatment, suggesting a more complex integration of volatiles from the two sources.

*D. rapae* was strongly attracted to odour of flowering *S. alba*, but preferred odour of flowers from *L. erysimi*-infested plants over odour of flowers from uninfested plants. This suggests that this *Brassica* specialist parasitoid can use volatile cues from the flowering parts of *L. erysimi*-infested plants. The cue may be a reduced concentration of floral volatiles or changes in the ratio of floral components that could potentially signal the presence of aphids. Alternatively it could be induced volatiles from the vegetative tissues supporting the flowers that were below the detection limits in our analysis, or a combination of the above. Thus, while aphid natural enemies showed modified responses to aphid-induced floral volatile inhibition, we did not find strong evidence that aphids would become less chemically apparent as a consequence. Although *D. rapae* may have an oviposition preference for *M. persicae* over *L. erysimi*, the parasitoid did not show an olfactory preference for plants infested with either aphid and this is in line with a previous study [42].

Flower chemistry and defensive chemistry have a common evolutionary origin [9], however most of the work relating plant defence and pollination has emphasised the dual role of floral chemistry in terms of attracting pollinators and reducing herbivory to reproductive parts [e.g. 43], [44], [45]. Our findings suggest a more intimate link between plant responses to insect herbivory, in this case by a phloem-feeder, and the biosynthesis of floral volatiles. They also reveal differences in the way specialist and generalist aphids interact with plant biochemistry, and this discovery may eventually help to illuminate how aphids avoid or manipulate plant defences.

## Materials and Methods

### Plants and insects

*Sinapis alba* seeds (Herbiseed, Twyford, UK) were germinated in a glass Petri dish on damp filter paper. Once the cotyledons began expanding (after three days) seedlings were transplanted into 8×8 cm pots with potting compost (Hasselfors Garden). Plants were placed in a glasshouse at 20±2°C between February and October with supplementary lighting (halogen) giving a minimum photoperiod of 16L∶8D and were watered daily. Flower buds began to appear after approximately 20 days. When the first flower buds began to open, herbivory treatments were applied as described below.

Colonies of mustard aphid *Lipaphis erysimi*, peach-potato aphid *Myzus persicae* and diamondback moth *Plutella xylostella* (started from naturally occurring summer migrants colonising Brassicaceae in Uppsala, Sweden) were maintained on oilseed rape, *Brassica napus* L.. The herbivores were reared in cages in a glasshouse at 20±2°C with a photoperiod of 16L∶8D with supplementary lighting.

Adults of the seven-spot ladybird *Coccinella septempunctata*, an important aphid predator [46] were collected from natural habitats close to Uppsala, Sweden (59°47′N and 17°39′E), and were reared in culture in cages with the cereal aphid *Rhopalosiphum padi* on barley and flowering *B. napus* as food at 21±1°C, a photoperiod of 16L∶8D, and relative humidity 60±10%. Aphid parasitoids, *Diaeretiella rapae*, were reared on *L. erysimi* on *B. napus*, under the same conditions as ladybirds, through at least two generations before use. Mummies were removed from the culture attached to leaf pieces and kept in a small emergence cage with honey solution (1∶1 in water) as food. Males and females emerged, but only females were used for experiments, and were 2–3 days old and assumed to be mated.

### Effects of herbivory on floral volatile production

#### Volatile collection and analysis

Volatiles emitted by *S. alba* flowers were collected by air entrainment [47]. The entrainment vessel consisted of a 350 ml modified glass beaker with an air inlet on the side, and an outflow at the top. The base of the vessel consisted of two aluminium plates, each with a notch in the middle that fitted around the stem. In this way only the flower and unopened buds were contained in the vessel. The plates were secured to the vessel with clips, and the vessel was held at flower height. A glass liner (Atas GL Intl., Veldhoven, Netherlands) containing Tenax TA (50 mg) was inserted through the outflow at the top of the vessel and connected to a pump. A positive pressure push-pull system was used, with charcoal-filtered air pushed in through a Teflon tube inserted through the inlet in the bottom of the vessel, at 600 ml/min and pulled out over the adsorbent at 300 ml/min. The difference in flow rates created a small positive pressure ensuring air from the laboratory did not enter the system. Before each entrainment all glass, Teflon material and filters (connected to a flow of nitrogen) were baked in an oven at 175°C for at least 16 h. Adsorbent tubes were baked for 16 h on a heating block at 230°C under a flow of nitrogen. The plants were watered daily at the base with 150 ml water during the entrainment period.

Collected volatiles were analysed by gas chromatography (GC) on a Hewlett Packard 6890 N (Agilent Technologies) GC-flame ionisation detector (FID), equipped with an HP-1 column (100% dimethyl polysiloxane, 50 m, 0.32 mm i.d. and 0.52 µm film thickness, J&W Scientific, USA), and fitted with an Optic-3 thermal desorption system (Atas GL Intl., Veldhoven, Netherlands). Internal standards, 600 ng of octane (IS-1) and 600 ng of dodecanal (IS-2) in 1 µl hexane were injected onto the Tenax containing the sample prior to desorption. The liner containing the Tenax with adsorbed volatiles was placed directly into the injector and volatiles were thermally desorbed starting at 40°C/0.5 min, and rising at 16°C/sec to 250°C. The GC temperature programme was 40°C/3 min, 5°C/min to 150°C/0.1 min, 10°C/min to 250°C/15 min, using hydrogen as carrier. The amount of each compound with Kováts retention index (RI) less than 1100 was calculated relative to the area of IS-1, while those with RI higher than 1100 were calculated relative to IS-2.

For tentative compound identification, a sample from each treatment was collected in the same way and analysed by coupled GC-mass spectrometry (GC-MS) using a Thermo Finnigan MAT 95 XP magnetic sector instrument coupled to a Finnigan Trace 2000 GC fitted with an Optic 400 thermal desorption system (Atas GL, UK) and a capillary GC column (DB-1, 100% dimethyl polysiloxane, 50 m, 0.32 mm i.d. and 0.52 µm film thickness, J & W Scientific). Ionization was by electron impact (70 eV, source temperature 220°C). The GC oven temperature was programmed at 30°C/5 min and 5°C/min to 250°C. A Tenax tube was inserted into the unit via the inlet liner and the volatiles released directly onto the GC column by thermal desorption (starting temperature of 20°C, rising at 16°C/sec to 220°C). Compounds were tentatively identified by comparison against a mass spectra library (NIST 05) and by comparison of mass spectra and retention indices with commercially available authentic standards.

(*E,E*)-α-Farnesene and (*E*)-ocimene were synthesized in high purity (>95% by GC) in two steps via functionalization of 3-methyl-3-sulpholene with geranyl bromide and 1-bromo-3-methylbut-2-ene respectively [48] and extrusion of sulphur dioxide using lithium aluminium hydride [49]. All other chemicals were purchased from Sigma-Aldrich, Sweden with the following purities: benzaldehyde >99%, 6-methyl-5-hepten-2-one 99%, (+)-sabinene 99%, β-pinene 99%, myrcene 90%, (*Z*)-3-hexenyl acetate 98%, benzyl alcohol 99%, benzyl acetate >99.7%, methyl salicylate 98%, *p*-anisaldehyde 98%, indole 99%, 2-undecanone >97%, benzyl isothiocyanate 98%, vanillin 99%, methyl vanillin 99%, 3,4,5-trimethoxy benzaldehyde 98%.

For confirmation of compound identity, peak enhancement co-injection was carried out with solvent extracts. Three solvent extracts of each treatment were collected by air entrainment, as described above, but using Porapak Q (50 mg, Alltech, USA) as the adsorbent. The compounds were eluted with redistilled dichloromethane (500 µl), and all samples from the same treatment were bulked and concentrated to 200 µl under nitrogen. Identifications were confirmed by GC peak enhancement with authentic samples with a temperature programme of 30°C/1 min, 5°C/min to 150°C/0.1 min, 10°C/min to 250°C/20 min, using hydrogen as carrier.

#### Effect of three different herbivores on floral volatiles

To investigate the effect of herbivory on vegetative tissues on production of floral volatiles in *S. alba*, plants that were about to enter anthesis were randomly allocated to one of four experimental treatments: 1.) undamaged control (UD), 2.) infestation with 200 mixed instar *L. erysimi* (LE), 3.) infestation with 200 mixed instar *M. persicae* (MP) or 4.) infestation with four third instar *P. xylostella* larvae (PX). These densities were chosen since they represent moderate to heavy levels of damage. The plants were individually isolated in perforated plastic bags (55×30 cm, Cryovac, UK).

To prevent the herbivores from feeding on flowers or unopened buds, flowers were separated from the vegetative parts. This was done by cutting an opening in the closed end of the bag and inserting the inflorescence and the two youngest fully expanded leaves. A piece of rubber foam was then placed around the stem under the second youngest leaves, and the bag was gently closed over the foam with a wire twist-tie. Herbivores were placed at the base of the plant, and the bag was closed with a rubber band around the pot. For undamaged control plants, the same isolation procedures were made, but no herbivores were placed on the plant. If any plant was damaged during the process it was discarded and replaced. Plants were placed in a greenhouse at 20±2°C with a photoperiod of 16L∶8D with supplementary lighting, on plastic dishes and watered daily at the base with 150 ml water.

Forty-eight hours after infestation, plants were transferred to a controlled environment chamber (21±1°C, 16L∶8D) for volatile collection. Two samples were collected: one spanning the period 48–72 h after infestation and one spanning 72–96 h after infestation. The four treatments were replicated five times in a randomised block design. The total amount of volatiles released 48–72 and 72–96 h after the start of damage were analysed by means of separate ANOVA, fitting treatment and block as categorical explanatory factors. In order to further analyse the changes in floral scent after herbivore attack a discriminant analysis using compositional data was carried out. First, the prevalent compounds (the benzenoids) were converted to compositional log-ratio data according to [50], using the compound 2-undecanone as a baseline reference. Then a discriminant function was fitted to these data to determine the separation between the treatments, and to determine the variables (compounds) responsible for the separation. Since the data were expressed as relative amounts (composition), interpretation should be carried out in terms of relative contribution of each compound to the blend as a whole. The analyses were carried out for the experiment at 48–72 hours after the start of damage and 72–96 hours after damage. This and all subsequent statistical analyses of floral volatiles were carried out using the R Environment (R Development Core Team, 2007).

#### Temporal pattern and aphid density dependence of floral volatile reduction by *L. erysimi*

The effect of *L. erysimi* density and time after infestation on the reduction in floral volatile production observed in the previous experiment was investigated. We manipulated *L. erysimi* density on plants at the beginning of the flowering stage to give four treatments: 1.) UD control; 2.) 50 mixed instar *L. erysimi* (AD50), 3.) 100 mixed instar *L. erysimi* (AD100) and 4.) 200 mixed instar *L. erysimi* (AD200). Infestation and volatile collection were performed as described above. A sample was collected every 24 h until 72 h after infestation.

Collections were replicated six times per treatment in a randomised block repeated-measures design. The total amount of volatiles was analysed by a repeated measures ANOVA fitting aphid density as a continuous explanatory variable with repeated measures across the continuous variable time. The repeated measures structure was analysed by fitting a split plot model with treatment (aphid number) as a continuous variable and block at the between subjects level. Time was fitted at the within subject (repeated measures) level. The amount of volatiles released (logarithmically transformed) was fitted as the response variable.

#### Effect of post-flowering infestation with *L. erysimi*

In the experiments above, aphid infestation occurred just after the onset of anthesis, when floral volatile production was commencing. In this experiment we addressed whether aphid feeding would shut down volatile production after it had already reached its natural level. The first flowers from two *S. alba* individuals were allowed to open after which the air-entrainment was set up with all plants still undamaged, but with the isolation bags already in place over the vegetative parts. A sample of volatiles was taken after 24 h to obtain a baseline reading for each experimental plant. One randomly chosen plant was then infested with 200 mixed instar *L. erysimi* (aphid damaged – AD) while the other was left undamaged (UD). The air entrainment was set up again, and samples were collected at 0–24 h and 24–48 h after the start of damage. This was repeated five times, giving five replicates for each treatment in a randomised block, repeated measures design.

Data were analysed by fitting a split plot model with treatment (UD or AD) and block as factors at the between-subject level. The baseline reading was fitted as a continuous covariate at the between-subject level. Time was fitted at the within subject (repeated measures) level. The amount of volatiles released (logarithmically transformed) was fitted as the response variable.

#### Effect of *L. erysimi* on number of *S. alba* flowers

A glasshouse experiment was carried out to determine whether a change in *S. alba* flower number after *L. erysimi* feeding could be responsible for the effect of the aphid on floral volatiles. Plants were enclosed in bags as described above and each plant was allocated randomly one of two treatments: UD or damaged by 200 *L. erysimi*, as in the volatile collections. After 72 hours the number of flowers on the main flower head was counted. A total of 10 replicate plants per treatment were assessed in a blocked design. The number of flowers was analysed by ANOVA, fitting block and treatment as explanatory factors.

### Effect of aphids on volatile production from vegetative tissues

To investigate the effect of aphid herbivory on volatile production from vegetative tissues, *S. alba* plants that were about to enter anthesis (the same growth stage from which floral volatiles were collected and that was used in olfactory bioassays) were randomly allocated to one of three experimental treatments: 1) undamaged control (UD) 2) infestation with 200 mixed instar *L. erysimi* (LE) 3) infestation with 200 mixed instar *M. persicae* (MP). The infestation was made by cutting an opening in the closed end of a perforated plastic bag (55×30 cm, Cryovac, UK) and enclosing the stem and leaves but excluding the inflorescence. A piece of rubber foam was then placed around the stem under the lowest leaves, and the bag was gently closed over the foam with a wire twist-tie. Aphids were placed at the base of the plant, and the bag was closed with a rubber band around the pot. For undamaged control plants, the same isolation procedures were made, but no aphids were placed on the plant. If any plant was damaged during the process it was discarded and replaced. Forty-eight hours after infestation, plants were transferred to a controlled environment chamber (21±1°C, 16L∶8D) for volatile collection which was done during the period 72–96 h after infestation in the same way as for the floral volatile collections. Treatments were replicated six times.

Collected volatiles were analysed and quantified by gas chromatography (GC) by the same procedure as for floral volatiles, using 1-nonene (5 ng) as internal standard. For tentative compound identification, a sample from each treatment was collected in the same way and analysed by coupled GC-mass spectrometry (GC-MS) using a an Agilent 7890 N (Agilent Technologies) GC coupled to an Agilent 5975 C mass selective detector (electron impact 70 eV). The GC was equipped with an HP-1 column (100% dimethyl polysiloxane, 50 m, 0.32 mm i.d. and 0.52 µm film thickness, J&W Scientific, USA), and fitted with an Optic 3 thermal desorption system (Atas GL Intl., Veldhoven, Netherlands). The liner containing the Tenax with absorbed volatiles was placed directly into the injector and volatiles were thermally desorbed starting at 30°C/0.5 min, and rising at 30°C/sec to 250°C. The GC temperature program was 30°C/4 min, 5°C/min to 150°C/0.1 min, 10°C/min to 250°C/15 min, using helium as carrier with a flow rate of 1.3 ml/min. Compounds were tentatively identified by comparison against a mass spectra library (NIST 08) and by comparison of mass spectra and retention indices with authentic standards. (*E,E*)-α-Farnesene was synthesized as described earlier. 4,8,12-Trimethyl-1,3,7,11-tridecatetraene (TMTT) was synthesized from (*E,E*)-farnesol by oxidation to its aldehyde followed by Wittig methylenation [51]. 3-butenyl isothiocyanate was synthesised as described in [52]. All other chemicals were purchased from Sigma-Aldrich, Sweden with the following purities: 6-methyl-5-hepten-2-one 99%, (*Z*)-3-hexenyl acetate 98%, hexyl acetate 99%, heptyl acetate >98%, 2-ethylhexyl acetate 99%, methyl salicylate 98%, nonyl acetate >97%, 2-undecanone >97%, benzyl isothiocyanate 98%, 2-tridecanone 99%.

The total amount of volatiles was compared among treatments using ANOVA, fitting treatment and block as categorical explanatory factors. The blends of were compared among treatments by converting the data to composition as described above (using hexyl acetate as a baseline) and using discriminant analysis as described above, including all compounds.

### Pollinator visits to *L. erisymi*-infested plants

Pollinator observations were carried out in fields surrounding the Department of Ecology, SLU, Uppsala, Sweden between April and June in 2008 and 2009. For each observation greenhouse-grown plants were randomly assigned to one of three treatments: undamaged (UD), damaged by 200 *L. erysimi* (Le) or damaged by 200 *M. persicae* (Mp). The plants were infested as described above and left for 48 h in the greenhouse. After this period the plants were taken to the field and placed in a line, with one metre separating each plant. Each line of plants was monitored visually by a single observer at a distance of three metres during four one-hour periods on the same day: 8:00–9:00, 11:00–12:00, 14:00–15:00 and 17:00–18:00. The position of plant treatments was alternated before each observation period. Every landing on the flower by different taxonomic groups of insects was recorded. The visits were summed across the different periods to give a total number of visits to each replicate plant. A total of ten replicates per treatment were carried out, five in 2008 and five in 2009. The total number of visits observed per day was analysed by ANOVA fitting treatment and year as explanatory factors, carried out using the R Environment (R Development Core Team, 2007). The overall numbers of visits was too low to allow separate analysis according to pollinator taxonomic group.

### Olfactory orientation of aphid natural enemies to floral volatiles

We tested whether aphid-induced changes in floral volatile production affects olfactory orientation by two important aphid natural enemies, both of which use aphid-induced volatiles in host location [23], [38] and can use flower resources as food. The experiments were designed to test the relative importance of volatile cues from flowering and vegetative plant parts, and comparative responses to plants infested with the different aphids species. Plants used as odour sources were enclosed in transparent polyester (PET) bags (Melitta Scandinavia, Sweden) (35×43 cm). For whole plant sources, a plant including its plastic pot and soil were enclosed in a bag sealed with a metal twist-tie above the soil. Where flower heads or green parts were separated, bags were sealed carefully around the stem using rubber foam and a twist-tie. A small hole was made through which a Teflon tube was inserted, sealed with labfilm (Parafilm) and connected to the olfactometer arm. A second hole was made in the opposite end of the bag with a Teflon tube connected through which air could enter and flow over the plant. The tube was 50 cm long to avoid re-collecting air in the direct vicinity of the odour sources. Aphid-damaged plants were infested with 200 aphids 72 h prior to the bioassays and aphids were not removed from the plant before the tests.

*C. septempunctata* were tested in a two-way airflow olfactometer with an airflow of 150 ml/min through each arm [53]. An adult ladybird was placed in the olfactometer for 10 minutes and its position recorded at 2 minute intervals. The observation frequency method [53] was used as it gives a reliable measure irrespective of whether the behaviour is characterized by frequent short visits or few long visits in the olfactometer arm. The accumulated number of observations in the arm zones after ten observations was regarded as one observation. If an insect did not move between three consecutive observations (was motionless) the replicate was discarded and a new one started with a fresh insect. Data were analysed with Wilcoxon matched pairs tests in the Statistica software (Statsoft Inc. 2005). Each experiment was replicated with 20–22 individual ladybirds, using five olfactometers simultaneously with the positions of the treatment arms alternating and with each olfactometer connected to separate plant individuals. In all experiments, plants were at the flowering stage. With *C. septempunctata* the following comparisons were carried out:

- Attraction to undamaged *S. alba*.
  1. Undamaged plant vs. clean air
  2. Flowers of undamaged plant vs. clean air
- Comparison of undamaged and *L. erysimi*-damaged *S. alba*
  1. Flowers of undamaged plant vs. flowers of *L. erysimi*-damaged plant
  2. Undamaged plant vs. *L. erysimi*-damaged plant
  3. Vegetative parts of undamaged plant vs. vegetative parts of *L. erysimi*-damaged plant
- Comparison of *L. erysimi*-damage with *M. persicae*-damage
  1. *Lipaphis erysimi*-damaged plant vs. *M. persicae*-damaged plant
  2. *Lipaphis erysimi*-damaged plant (vegetative parts) vs. *M. persicae*-damaged plant (vegetative parts).
  3. Flowers of *L. erysimi*-damaged plant vs. flowers of *M. persicae*-damaged plant.

*D. rapae* were tested using a two-way airflow olfactometer described by Glinwood et al. [54] with an airflow of 125 ml/min through each arm. A female parasitoid was placed in the olfactometer and, during 10 minutes, the amount of time spent by the insect in the arms was recorded. Twenty parasitoids were used in each experiment. After every five replicates, plant odour sources were replaced by different plants of the same treatment, and the spatial positions of the treatments alternated. The mean amount of time spent by parasitoids in the arms was analysed using Wilcoxon matched pairs tests in the Statistica software (Statsoft Inc. 2005). The following comparisons were carried out:

1. Undamaged plant vs. clean air
2. Flowers of undamaged plant vs. flowers of *L. erysimi*-damaged plant
3. *Lipaphis erysimi*-damaged plant vs. *M. persicae*-damaged plant
4. Flowers of *L. erysimi*-damaged plant vs. flowers of *M. persicae*-damaged plant.
